# Second symptomatic COVID-19 infections in patients with an underlying monoclonal gammopathy

**DOI:** 10.1038/s41408-022-00752-z

**Published:** 2022-11-24

**Authors:** Saurabh Zanwar, Matthew Ho, Francis K. Buadi, Sikander Ailawadhi, Jeremy Larsen, Leif Bergsagel, Moritz Binder, Asher Chanan-Khan, David Dingli, Angela Dispenzieri, Rafael Fonseca, Morie A. Gertz, Wilson Gonsalves, Ronald S. Go, Suzanne Hayman, Prashant Kapoor, Taxiarchis Kourelis, Martha Q. Lacy, Nelson Leung, Yi Lin, Eli Muchtar, Vivek Roy, Taimur Sher, Rahma Warsame, Amie Fonder, Miriam Hobbs, Yi L. Hwa, Robert A. Kyle, S. Vincent Rajkumar, Shaji Kumar

**Affiliations:** 1 Department of Internal Medicine, Mayo Clinic, Rochester, MN USA; 2 Division of Hematology, Department of Internal Medicine, Mayo Clinic, Rochester, MN USA; 3 Division of Hematology, Mayo Clinic, Jacksonville, FL USA; 4 Division of Hematology, Mayo Clinic, Scottsdale, AZ USA

**Keywords:** Epidemiology, Diagnosis

Patients with hematologic malignancies, including multiple myeloma (MM), light chain (AL) amyloidosis, smoldering MM (SMM) and monoclonal gammopathy of undetermined significance (MGUS), have disproportionately high rates of COVID-19 infection and associated mortality [1, 2]. Immune paresis is a common feature in dysproteinemias and has been demonstrated to be an independent risk factor for severity of COVID-19 infection in patients with MGUS [3]. In a matched control study of patients with MM and MGUS compared with healthy controls, the risk of breakthrough infections after completion of vaccination was around 3% [4]. Ongoing myeloma-directed treatment further induces immune suppression increasing the risk for severe COVID-19 infections [5–7]. While inferior immune response to COVID-19 vaccination have been described in patients with monoclonal gammopathies [8], information on the rate and severity of COVID-19 infections in patients who do demonstrate immunogenicity post infection or vaccination is limited. Additionally, rates and clinical course of second symptomatic COVID-19 infections in patients with monoclonal gammopathy in relation to prior antibody testing has been previously described.

After institutional review board approval, patients with MGUS, SMM, MM, or AL amyloidosis evaluated at Mayo Clinic Rochester, Arizona, and Florida between 12/01/2019 and 8/31/2021 were screened. Patients with a positive polymerase chain reaction test for SARS-CoV-2 and either a spike antibody (Ab) or a nucleocapsid Ab tested were included. Spike Ab was reported quantitatively (positive result >0.8 IU/mL per laboratory standard) and nucleocapsid Ab testing was reported as either positive or negative. For this study, we defined “fully vaccinated” as 2 doses of Pfizer or Moderna mRNA vaccine or 1 dose of the Janssen vaccine based on the prevalent CDC recommendation during the study period. Severe COVID-19 infection was defined using established criteria [9]. Patients with a 2nd symptomatic COVID-19 infection had an interim negative COVID-19 test result with complete clinical recovery.

Out of 19,943 patients with an underlying monoclonal gammopathy, 483 patients (2.4%) had a documented positive COVID-19 PCR test and 101 patients had COVID-19 antibody testing performed [spike Ab = 54, nucleocapsid Ab = 47] (Supplementary Fig. 1). The baseline characteristics of these 101 patients are depicted in Table 1. The cohorts of patients with an Ab test performed (*n* = 101) versus those who did not have an antibody testing done (*n* = 382) were largely comparable for baseline characteristics, vaccination status and the severity of 1st COVID-19 infection (Supplementary Table 1)*.* Majority (84%) of the patients were unvaccinated at the time of 1st COVID-19 infection. By the end of the follow-up period, 68% of the patients were fully vaccinated. Data regarding vaccination status are depicted in Supplementary Table 2.

**Table 1:** Baseline characteristics for the study cohort.

Characteristics	*N* (%)
Total number of patients	101 (100%)
MGUS	56 (55%)
SMM	3 (3%)
MM	39 (39%)
AL amyloidosis without coexisting MM	3 (3%)
Age at time of COVID-19 diagnosis, years	70 (37–89)
Female sex	28 (28%)
High risk FISH in patients with MM	17/38 (45%)
ISS stage in patients with MM	
Stage 1	10/37 (27%)
Stage 2	16/37 (43%)
Stage 3	11/37 (30%)
Number of lines of treatment prior to COVID-19 diagnosis	
MGUS	0 (0–1)
SMM	0
MM	2 (0–13)
AL amyloidosis	3 (2–4)
Anti-CD38 therapy within 6 months of the 1st COVID-19 diagnosis in MM/AL	14/42 (33%)
ASCT at any time prior to COVID-19 diagnosis in MM/AL	25/42 (60%)
ASCT within 6 months of COVID-19 diagnosis in MM/AL	4/42 (10%)
CAR-T cell therapy any time prior to COVID-19 diagnosis in MM	2/39 (5%)
CAR-T cell therapy within 6 months of COVID-19 diagnosis	0
Immunoparesis within 3 months of COVID-19 diagnosis	51/62 (82%)

*AL* light chain amyloidosis, *ASCT* autologous stem cell transplant, *CAR-T* chimeric antigen receptor T cell, *COVID-19* Coronavirus disease 2019, *FISH* fluorescent in situ hybridization, *ISS* international staging system, *MGUS* monoclonal gammopathy of undetermined significance, *MM* multiple myeloma, *SMM* smoldering multiple myeloma

## Seropositivity in patients unvaccinated at the time of 1st COVID-19 infection

Of the 85 unvaccinated patients at the time of first infection, 63 (74%) developed a positive COVID-19 Ab after the 1st symptomatic infection (spike Ab *n* = 38, nucleocapsid Ab *n* = 25), Supplementary Table 3. Out of these 63 patients, 7 (11%) developed a second symptomatic COVID-19 infection at a median time of 9.9 (range 2.9–19.6) months from first COVID-19 infection and 6 (range 2–18.7) months from the time of antibody testing. All 7 patients had seropositivity prior to the 2nd infection; 6 of these 7 patients had a positive spike Ab [values: 66, 115, 190, and >250 IU/ml (*n* = 3)] and 1 had a positive nucleocapsid Ab. In those without seropositivity (*n* = 22/85), 4 patients (18%) developed a 2nd symptomatic infection, 1 of which was severe.

## Seropositivity in patients unvaccinated at the time of 1st COVID-19 infection

To assess seropositivity after vaccination in patients without a prior COVID-19 infection, spike Ab was used as nucleocapsid Ab can be negative after mRNA vaccination. Among the vaccinated patients at the time of first infection (*n* = 16), 6 had a spike Ab tested before 1st COVID-19 infection at a median of 65 (range 16–109) days from vaccination (Supplementary Table 3). Of these, 5 (83%) demonstrated a positive spike Ab response to the vaccination. Despite seropositivity, all 5 developed COVID-19 and 2 patients with positive spike Ab developed a severe 1st COVID-19 infection (without mortality) at 84 days and 99 days from vaccination, respectively [spike Ab level was 145 IU/mL and > 250 IU/mL, respectively]. None of the vaccinated and seropositive patients (*n* = 10) developed a 2nd symptomatic COVID-19 infection. Comparatively, out of the 63 unvaccinated patients with seropositivity after 1st COVID-19 infection, 7/63 (11%) had developed a 2nd symptomatic infection (*p* = 0.59).

## Second symptomatic COVID-19 infection in relation to antibody testing

**Fig. 1:**
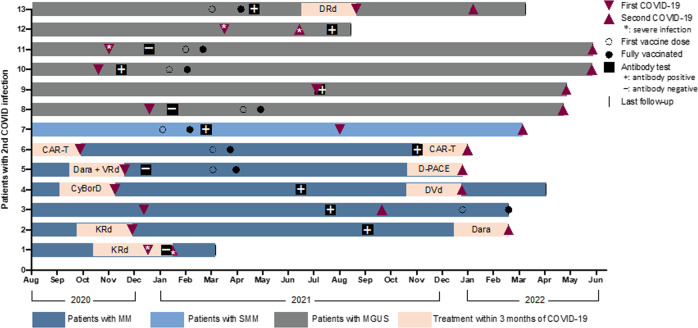
Second COVID-19 infections. The figure demonstrates a timeline of second COVID-19 infection in patients with monoclonal gammopathy in relation to antibody status and treatment administered.

Out of the 101 patients, 13 (13%) developed a 2nd symptomatic COVID-19 infection. Details of the timeline for second infections in relation to vaccination, antibody testing and ongoing treatment are depicted in Fig. 1. The majority of 2nd COVID-19 were mild (11/13, 85%) and the 2 patients (15%) with a severe 2nd infection were both unvaccinated. The median time to 2nd infection was 6.3 months (range 1–19 months) from the 1st COVID-19 infection. Rate of 2nd infection was 21% (6/28) in unvaccinated patients compared to 9.6% (7/73) in patients after completion of initial vaccination series (*p* = 0.11). Six out of the 7 vaccinated patients had received a third dose of vaccination prior to the 2nd infection.

Of the 42 patients with MM/AL, 25 patients were in remission and 1 out of these 25 (4%) patients developed a 2nd COVID-19 infection. Compared to patients in remission, 5 (29%) of the 17 patients with MM/AL not in remission developed a 2nd infection (*p* = 0.03). Details of ongoing treatment for MM prior to the 2nd infection are depicted in Fig. 1. Among the 7 patients with MGUS/SMM, all 4 patients with immunoglobulin testing performed at the time of 2nd infection had immune paresis. Among the 382 patients in whom an antibody test was not performed, 22 (6%) patients developed a second infection out of which 3 (14%) were severe. Details of second infection in the of patients where an antibody test was not performed are depicted in Supplementary Fig. 2.

Patients with monoclonal gammopathy demonstrate an inferior seroconversion to COVID-19 vaccination but the rate and severity of 2nd infections in the context of prior seropositivity have not been described [8, 10]. To address this issue, we focused on patients who had an antibody test performed. Majority of the patients in our cohort demonstrate seropositivity after COVID-19 infection and/or vaccination. Despite this, the incidence of a second symptomatic COVID-19 infection was substantial and a short time to second COVID-19 infection (median of 6 months) points toward a rapidly waning immunity. Similarly, patients with a documented positive spike antibody before the 1st COVID-19 infection still went on to develop COVID-19, including severe infections. This further highlights the need for booster doses in this patient population.

In our study, the risk of a second infection was 9.6% in patients who had already experienced a COVID-19 infection and were fully vaccinated prior to the second infection. This occurred despite majority of the patients having received a 3rd dose of the mRNA vaccine. Even though recurrent infections were noted despite vaccination, there was a distinct trend towards a lower rate of 2nd infections in the vaccinated population. Notably, all severe 2nd COVID-19 infections occurred in patients that were unvaccinated at the time of the second infection, and this should serve as a reaffirmation of the importance of vaccination even in patients with a prior COVID-19 infection. These data continue to highlight the importance of vaccination even in patients who have had previous COVID-19 infection and a documented seropositivity.

This study has limitations, most prominent of which is the random nature in which patients underwent antibody testing, potentially leading to a selection bias. To ensure there was no systemic bias introduced while selecting this cohort, we compared the cohort of patients who had an antibody test performed versus those who did not undergo testing, and these were largely comparable. Another limitation is the type of antibody tested and the timing of testing in relation to the infection or vaccination. Despite these limitations, this study offers valuable information on rates and clinical characteristics of 2nd COVID-19 infections.

In conclusion, patients with an underlying MM, AL, or MGUS demonstrate a substantial risk for second symptomatic COVID-19 infections suggesting a rapidly waning immunity. Most of the 2nd COVID-19 infections were mild, with severe infections restricted to the unvaccinated population. Patients in whom underlying MM or AL was in remission was protective against a 2nd infection.

## Supplementary information

Supplement

## Data Availability

There is no relevant data to disclose.
