# Supplementary material for: Second symptomatic COVID-19 infections in patients with an underlying monoclonal gammopathy
Source: Blood Cancer J. 2022 Nov 24;12(11):160. doi: 10.1038/s41408-022-00752-z (PMC9686231; doi:10.1038/s41408-022-00752-z)
Supplement: Supplementary file 1 — Supplement [file 41408_2022_752_MOESM1_ESM.docx]

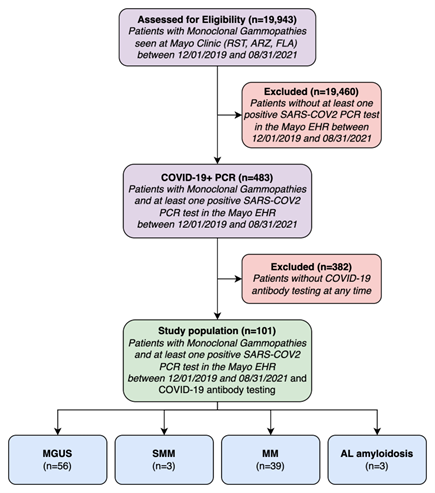


**Supplementary Figure 1: Selection of the Patient Population.** The Mayo Clinic (Rochester, Arizona, and Florida) electronic health record was screened and 19,943 consecutive patients with monoclonal gammopathy (i.e., MGUS, SMM, MM, and AL amyloidosis) who were seen between 12/01/2019 and 08/31/2021 were identified. From this initial cohort, 483 patients also had at least one positive *SARS-COV2* PCR test between 12/01/2019 and 08/31/2021, and a further 101 patients who also had antibody testing were included in subsequent analyses.

**Timing of Antibody testing:** Out of the 101 patients, 20 patients had the antibody test performed prior to the first COVID-19 infection, 81 patients had the antibody test performed after the 1^st^ COVID-19 infection, out of which 1 was after the 2^nd^ COVID-19 infection. Only one antibody test result was available for all patients.

| **Supplementary Table 1.** Comparison of Baseline Characteristics of Patients a COVID-19 infection with and without an Antibody test performed | | | |
| --- | --- | --- | --- |
|  | Median (range) or N (%) | | P-value |
|  | Ab test | No Ab test |  |
| Total number of patients | 101 (100%) | 382 (100%) |  |
| MGUS | 56 (55%) | 234 (61%) | 0.29 |
| SMM | 3 (3%) | 3 (1%) | 0.11 |
| MM | 39 (39%) | 135 (35%) | 0.54 |
| AL amyloidosis without coexisting MM | 3 (3%) | 10 (3%) | 0.74 |
| Age at time of COVID-19 diagnosis, years | 70 (37–89) | 70 (23–98) |  |
| **Female sex** | **28 (28%)** | **161 (42%)** | **< 0.01** |
| High risk FISH in patients with MM | 17/38 (45%) | 53/109 (49%) | 0.68 |
| ISS stage in patients with MM |  |  |  |
| Stage 1 | 10/37 (27%) | 48/113 (42%) | 0.094 |
| Stage 2 | 16/37 (43%) | 34/113 (30%) | 0.14 |
| Stage 3 | 11/37 (30%) | 31/113 (27%) | 0.79 |
| Number of lines of treatment prior to COVID-19 diagnosis |  |  |  |
| MGUS | 0 (0–1) | 0 (0–6) |  |
| SMM | 0 | 0 |  |
| MM | 2 (0–13) | 2 (0–10) |  |
| AL amyloidosis | 3 (2–4) | 1 (0–3) |  |
| Anti-CD38 within 6 months of COVID-19 diagnosis | 16/99 (16%) | 51/380 (13%) | 0.48 |
| ASCT within 6 months of COVID-19 diagnosis | 4/100 (4%) | 13/381 (3%) | 0.76 |
| CAR-T cell any time prior to COVID-19 diagnosis | 2/100 (2%) | 6/380 (2%) | 0.67 |
| CAR-T cell within 6 months of COVID-19 diagnosis | 0 | 2/380 (1%) | 1 |
| Immunoparesis within 3 months of COVID-19 diagnosis | 51/62 (82%) | 161/208 (77%) | 0.48 |
| COVID vaccine status at time of first COVID-19 infection |  |  |  |
| Non-vaccinated | 85 (84%) | 344 (90%) | 0.095 |
| Partially vaccinated | 7 (7%) | 13 (3%) | 0.15 |
| Pfizer (1 dose) | 3 (3%) | 7 (2%) | 0.44 |
| Moderna (1 dose) | 4 (4%) | 6 (2%) | 0.23 |
| Fully vaccinated | 9 (9%) | 25 (7%) | 0.39 |
| Pfizer (at least 2 doses) | 4 (4%) | 20 (5%) | 0.8 |
| Moderna (at least 2 doses) | 4 (4%) | 5 (1%) | 0.096 |
| J&J (at least 1 dose) | 1 (1%) | 0 | 0.21 |
| COVID vaccine status at time of second COVID-19 infection |  |  |  |
| Non-vaccinated | 6/13 (46%) | 7/16 (44%) | 1 |
| Partially vaccinated | 0 | 0 | 1 |
| Fully vaccinated | 7/13 (54%) | 9/16 (56%) | 1 |
| Pfizer (at least 2 doses) | 4/13 (31%) | 6/16 (38%) | 1 |
| Moderna (at least 2 doses) | 3/13 (23%) | 2/16 (13%) | 0.63 |
| J&J (at least 1 dose) | 0 | 1/16 (6%) | 1 |
| COVID-19 vaccine status at time of follow-up |  |  |  |
| Non-vaccinated | 27 (27%) | 122 (32%) | 0.31 |
| Fully vaccinated | 69 (68%) | 243 (64%) | 0.38 |
| Partially vaccinated | 5 (5%) | 17 (4%) | 0.79 |
|  |  |  |  |
| Ab: antibody; AL: amyloid light chain; ASCT: autologous stem cell transplantation; CAR-T: chimeric antigen receptor T-cell therapy; MGUS: monoclonal gammopathy of undetermined significance; MM: multiple myeloma; SMM: smoldering multiple myeloma | | | |

| **Supplementary Table 2: COVID-19 vaccination status among patients with antibody testing performed** | |
| --- | --- |
| Vaccination | N (%) |
| Total number of patients | 101 (100%) |
| COVID vaccine status at time of first COVID-19 infection |  |
| Unvaccinated | 85 (84%) |
| Partially vaccinated | 7 (7%) |
| Pfizer (1 dose) | 3 (3%) |
| Moderna (1 dose) | 4 (4%) |
| Fully vaccinated | 9 (9%) |
| Pfizer (at least 2 doses) | 4 (4%) |
| Moderna (at least 2 doses) | 4 (4%) |
| J&J (at least 1 dose) | 1 (1%) |
| COVID vaccine status at time of second COVID-19 infection |  |
| Unvaccinated | 6/13 (46%) |
| Partially vaccinated | 0 |
| Fully vaccinated | 7/13 (54%) |
| Pfizer (at least 2 doses) | 4/13 (31%) |
| Moderna (at least 2 doses) | 3/13 (23%) |
| J&J (at least 1 dose) | 0 |
| COVID-19 vaccine status at time of follow-up |  |
| Unvaccinated | 27 (27%) |
| Partially vaccinated | 5 (5%) |
| Pfizer | 4 (4%) |
| Moderna | 1 (1%) |
| Fully vaccinated | 69 (68%) |
| Pfizer (at least 2 doses) | 42 (42%) |
| Moderna (at least 2 doses) | 25 (25%) |
| J&J (at least 1 dose) | 2 (2%) |
| Ab: antibody; J&J: Janssen vaccine | |

| **Supplementary Table 3**. Seropositivity in relation to timing of vaccination | | | | | | |
| --- | --- | --- | --- | --- | --- | --- |
| Parameter | Positive | Negative | Spike Ab  >250 IU/ml | Severe infection | Mortality from 1^st^ COVID-19 infection | 2^nd^ COVID-19 infection |
| **Antibody tested (n=101)** | | | | | | |
| Spike Ab (n=54) | 52 | 2 | 38 | 14 | 1 | 8 |
| Nucleocapsid Ab  (n=47) | 26 | 21 | NA | 12 | 2 | 5 |
| **Unvaccinated Patients: Antibody tested after 1^st^ infection (n=85)** | | | | | | |
| Spike Ab (n=39) | 38 | 1 | 30 | 9/38 (+ Ab)  0/1 (– Ab) | 0 | 6/38 (+ Ab)  0/1 (– Ab) |
| Nucleocapsid Ab (n=46) | 25 | 21 | NA | 4/25 (+ Ab)  7/21 (­– Ab) | 1/25 (+ Ab)  1/21 (– Ab) | 1/25 (+Ab)  4/21 (- Ab) |
| **Vaccinated Patients: Ab tested before 1^st^ infection (n=6)** | | | | | | |
| Spike Ab  (n=6) | 5 | 1 | 3/6 | 2/5 (+ Ab)  0/1 (– Ab) | 0/5 (+ Ab)  0/1(– Ab) | 2/5 (+ Ab)  0/1(– Ab) |
| **Vaccinated Patients: Ab tested after 1^st^ infection (n=10)** | | | | | | |
| Spike Ab  (n=9) | 9 | 0 | 5 | 3 | 1 | 0 |
| Nucleocapsid (n=1) | 1 | 0 | NA | 1 | 0 | 0 |
| +Ab: positive antibody; -Ab: negative Ab; Ab: neutralizing antibody | | | | | | |

| **Supplementary Table 4. Severity of COVID-19 infection in patients with an antibody study performed** | |  |
| --- | --- | --- |
| Infection | N (%) |  |
| Total number of patients | 101 (100%) |  |
| Number of COVID infections |  |  |
| One | 101 (100%) |  |
| Two | 13 (13%) |  |
| Severity of first COVID-19 infection |  |  |
| Asymptomatic/Mild | 56 (55%) |  |
| Moderate | 19 (19%) |  |
| Severe | 26 (26%) |  |
| Death | 2 (2%) |  |
| Severity of second COVID-19 infection |  |  |
| Mild | 11/13 (85%) |  |
| Moderate | 0 |  |
| Severe | 2/13 (15%) |  |
| Death | 0 |  |
| Underlying Diagnosis and 2^nd^ infection | N=13 |  |
| MGUS | 6 (46%); 1 severe | |
| MM | 6 (46%); 1 severe | |
| SMM | 1 (8%) | |
| AL | 0 | |
| AL: light chain amyloidosis; MGUS: monoclonal gammopathy of undetermined significance; MM: multiple myeloma; SMM: smoldering multiple myeloma | | |

**Supplementary Figure 2:** Timeline of infection in ongoing treatment among patients with a Second Symptomatic COVID-19 Infection who did not undergo antibody testing. CyBorD: cyclophosphamide, bortezomib, dexamethasone; DPd: daratumumab, pomalidomide, dexamethasone, RMx: lenalidomide maintenance
